# Biomass Cellulose-Derived Carbon Aerogel Supported Magnetite-Copper Bimetallic Heterogeneous Fenton-like Catalyst Towards the Boosting Redox Cycle of ≡Fe(III)/≡Fe(II)

**DOI:** 10.3390/nano15080614

**Published:** 2025-04-16

**Authors:** Qiang Zhao, Jiawei Yang, Jiayi Xia, Gaotian Zhao, Yida Yang, Zongwei Zhang, Jing Li, Fang Wei, Weiguo Song

**Affiliations:** 1 College of Science, Civil Aviation University of China (CAUC), Tianjin 300300, China; 2 College of Aerospace Engineering, Civil Aviation University of China (CAUC), Tianjin 300300, China; 3 Laboratory of Molecular Nanostructure and Nanotechnology, Institute of Chemistry, Chinese Academy of Sciences, Beijing 100190, China; 4 Science and Technology Innovation Research Institute, Civil Aviation University of China (CAUC), Tianjin 300300, China

**Keywords:** heterogeneous Fenton-like catalyst, biomass, dye removal, wastewater treatment, catalytic mechanism

## Abstract

To degrade high-concentration and toxic organic effluents, we developed Fe-Cu active sites loaded on biomass-source carbon aerogel (CA) to produce a low-cost and high-efficiency magnetic Fenton-like catalyst for the catalytic oxidative decomposition of organic pollutants. It exhibits excellent performance in catalytic Fenton-like reactions for RhB removal at an ultrahigh initial concentration of up to 1000 ppm. To be specific, Fe_3_O_4_ and Cu nanoparticles are generated in situ on a mesoporous CA support, denoted as an Fe_3_O_4_-Cu/CA catalyst. Experimentally, factors including initial dye concentration, catalyst dosage, H_2_O_2_ dosage, pH, and temperature, which significantly influence the oxidative degradation rate of RhB, are carefully studied. The RhB (1000 ppm) degradation ratio reaches 93.7% within 60 min under low catalyst and H_2_O_2_ dosage. The catalyst also shows slight metal leaching (almost 1.4% of total Fe and 4.0% of total Cu leached after a complete degradation of 25 μmol RhB under conditions of 15 mg catalyst dosage, 20 mL RhB solution (600 ppm), and 200 μL 30 wt% H_2_O_2_ dosage, at pH of 2.5, at 40 °C), good catalytic activity for degrading organic pollutants, excellent reusability, and good catalytic stability (the degradation ratio is nearly 82.95% in the 8th cycle reaction). The synergistic effect between Fe and Cu species plays a vital role in promoting the redox cycle of Fe(III)/Fe(II) and enhancing the generation of ·OH. It is suitable for ultrahigh-concentration organic pollutant degradation in practical wastewater treatment applications.

## 1. Introduction

With the rapid development of global industrialization, the discharge of wastewater with different kinds of pollutants has gradually affected water ecological security and human health, which is also receiving considerable attention in society. Developing environmental remediation technologies to eliminate water contaminants is urgent in this case. Fenton or Fenton-like reaction is one of the most powerful advanced oxidation process (AOP) methods that can generate several kinds of reactive oxygen species (ROS) with ultrahigh oxidation potential (·OH: *E*_ox_ = +2.7 eV) from H_2_O_2_, which has been widely employed to degrade organic pollutants [1,2,3,4].

Currently, iron-based heterogeneous Fenton-like processes have been widely investigated for wastewater treatment due to their wide availability, stable catalytic activity, and convenient catalyst recycling [5,6]. Among these heterogeneous Fenton-like catalysts, Fe_3_O_4_ magnetic nanoparticles have attracted much attention for their multi-valence Fe sites promoting the redox cycle of Fe(III)/Fe(II) [7,8] for the generation of ROS. However, the use of these Fe_3_O_4_-based catalysts for the application of removing organic pollutants with high initial concentrations from wastewater is still a great challenge for environmental remediation. According to the literature, the Fe(II)/H_2_O_2_ reaction is the key process of generating hydroxyl radicals (·OH), whereas the Fe(III)/H_2_O_2_ reaction is the rate-limiting process of the regeneration of Fe(II) in the redox cycle of Fe(III)/Fe(II), which is much slower than the former reaction [9,10]. This may hinder the continuous mass production of ·OH in conventional iron-based heterogeneous Fenton-like systems.

Additionally, the surface area may decrease significantly when the aggregation of iron-based nanocrystals occurs in catalysts. The valence states of Fe sites probablychange when catalytic reaction happens. As a result, the iron-based nano-catalyst experiences a gradual decrease in catalytic reactivity. To enhance catalytic activity and stability, the immobilization of magnetite nanoparticles onto carbonaceous supports with high chemical stability has been proven to be a simple and effective method [11].

Carbon aerogel materials exhibit many advantages, such as abundant pore structure, high specific surface area, good thermal and chemical stability, favorable adsorption ability, tunable surface chemistry and microstructure, and environmental compatibility [12,13]. It can be a good candidate for catalyst support, which effectively stabilizes Fe_3_O_4_ nanoparticles and prevents the aggregation of nanoparticles [14,15]. Unfortunately, the Fe-based heterogeneous Fenton-like catalyst still faces the problem of hindered regeneration of Fe(II) in the redox cycle of Fe(III)/Fe(II).

Copper is also a promising active site for heterogeneous Fenton-like catalysts due to its low cost, good catalytic effect, and high utilization [16,17]. Sun et al. identified the critical roles of coexisting Cu(0), Cu(I), and Cu(II) species in the core–shell Cu/SiO_2_ catalysts in which high-valence Cu species are stepwise reduced at different temperatures for RhB degradation [18]. They proposed that Cu(I) acts as the primary active species with the highest efficiency in generating ·OH. Subsequently, they prepared an alumina-supported bimetallic Fe/Cu catalyst via the sol–gel method, which exhibited high performance in catalytic degradation of nitrobenzene, also revealing the synergistic effect between Fe and Cu species. Cu played a vital role in promoting the reduction of Fe(III) to Fe(II) [19]. Similarly, Song et al. manipulated the structure of Fe-oxo nodes in Fe-MOFs (MIL-88B), employing Cu(I) species for mixed-valence states with highly improved Fenton-like performance, in which system Cu(I) facilitates the conversion between Fe(II) and Fe(III), inducing the formation of a larger amount of stable Fe(II) [20]. This catalyst requires an additional H_2_/Ar reduction process at a high temperature, which increases its production costs. Some other researchers have reported that Cu-doped Fe_3_O_4_ not only alters the charge density of Fe_3_O_4_ to increase the adsorption of pollutants, but also improves the interfacial electron transfer through the redox cycle of Fe(III)/Fe(II) and Cu(II)/Cu(I) in the presence of H_2_O_2_ [21]. Shen et al. synthesized the biochar-supported magnetite/copper catalyst (Fe_3_O_4_/Cu/BC) by a one-step pyrolysis method using Fenton sludge as an iron source, which exhibited excellent Fenton catalytic activity and reusability for RhB degradation [22]. However, the sludge-derived catalyst support composition is complex, which may bring unknown factors into controlling product uniformity. Mao et al. also reported that Cu can be employed to enhance the efficiency of light utilization alongside Fe in their Fenton photocatalyst denoted as Fe/Cu-PPAO for the application of tetracycline hydrochloride removal [23]. Therefore, Cu species are important in bimetallic Fe/Cu-based Fenton-like catalysts for enhancing their catalytic performance.

Herein, we put forward a magnetic bimetallic Fe_3_O_4_-Cu/CA heterogeneous Fenton-like catalyst employing corn-cob (CC)-extracted cellulose-derived carbon aerogel as support. Biomass like corn-cob should be a sort of good carbonaceous source material rather than conventional coal or other fossil resources. It shows net-zero carbon emissions when utilized in modern industry. Lignocellulose is abundant in biomass, which can also be well-treated as raw materials to produce cellulose. To be specific, we proposed that CC-extracted cellulose aerogel (CC-CA) acts as a carbon aerogel (CA) support precursor. It produces lower greenhouse gas emissions. It is gradually transformed into carbon during pyrolysis in N_2_ flow, which also provides a reducing atmosphere for generating Fe(II) and Cu(0) species from their higher-valence ions, including Fe(III) and Cu(II). Fe_3_O_4_-Cu/CA catalysts with different Fe/Cu ratios are synthesized at a moderate pyrolysis temperature in order to screen out the optimal catalyst through catalytic degradation performance comparison. We also investigated its morphology, textual property, element composition, pore structure, crystal patterns, magnetic property, surface chemistry, carbonized structure, and surface chemical states. The Fenton catalytic performance of Fe_3_O_4_-Cu/CA was investigated through the RhB degradation reaction to evaluate its catalytic activity under several fundamental conditions, including substrate concentration, catalyst dosage, H_2_O_2_ dosage, pH, and temperature. Its reusability and stability were also investigated. We verified the influence of co-existing ions on the Fe_3_O_4_-Cu/CA catalytic Fenton-like system. The active oxidative species are identified through ROS quenching and radical capture experiments. The catalytic oxidation mechanism of Fe_3_O_4_-Cu/CA is also discussed. The results show that Fe_3_O_4_-Cu/CA can be used in high-efficiency Fenton reactions for eliminating ultrahigh-concentration organic contaminants with excellent reusable performance.

## 2. Materials and Methods

### 2.1. Materials and Reagents

The chemical reagents, including urea (CH_4_N_2_O, AR), ethanol, C_2_H_5_OH (AR), congo red (CR, AR), coomassie brilliant blue G250 (CBB, AR), basic fuchsin (BF, AR), rhodamine B (RhB, AR), methylene blue (MB, AR), and methyl orange (MO, AR), were purchased from Sinopharm Chemical Reagent (Beijing, China) and InnoChem Science & Technology Co., Ltd. (Beijing, China). Cellulose, NaOH (AR, 99%), HCl (AR, 37%), Fe(NO_3_)_3_·9H_2_O (AR, 98.5%), Cu(NO_3_)_2_·3H_2_O (AR, 99%), CHCl_3_ (AR, 99%), NaClO_2_ (AR, 80%), H_2_O_2_ (AR, 30 wt%), furfuryl alcohol (AR, 98%), CH_3_OH (AR, 99%), 2,2,6,6-Tetramethylpiperidine (GC, 98%), tetracycline hydrochloride (96%), and oxytetracycline hydrochloride (95%) were purchased from Aladdin Scientific Corporation (Shanghai, China) and local chemical reagent companies (Tianjin, China). The 5,5-dimethyl-1-pyrrolidine-N-oxide (DMPO, AR) and 2,2,6,6-tetramethylpiperidine (TEMP, AR) were purchased from Sigma-Aldrich Trading Co., Ltd. (Shanghai, China). The dry corn-cob was purchased from a local market and crushed with a grinder, denoted as CC in the following text. The water used in this article was distilled water produced by the laboratory. All the chemicals were used as received without further purification.

### 2.2. Preparation of Fe_3_O_4_-Cu/CA

The preparation of the CC-extracted cellulose was carried out as follows: CC (20 g) was placed in a three-necked flask, and 1000 mL of 5 wt% NaOH solution was added, with magnetic stirring at 80 °C in an oil bath for 3 h. The sediment was washed with deionized water until the supernatant was neutral. Then, the sediment was placed in a flask accompanied by 15 g of NaClO_2_, 5 mL of acetic acid, and 1000 mL of deionized water, stirring in a flask that was kept warm in an oil bath at 80 °C for 3 h. Subsequently, the product was separated and washed with deionized water until it was neutral. Then, the product was dispersed into a 2 wt% hydrochloric acid solution, stirring magnetically for 6 h to remove impurities such as inorganic salts from the cellulose. The obtained cellulose was washed until neutral and then dried to obtain cellulose powder (CC-extracted cellulose).

The preparation of the CC-CA was carried out as follows: The alkaline solution containing 7 g of NaOH, 12 g of urea, and 81 mL of deionized water was prepared. A total of 5 g of CC-extracted cellulose powder was added to the alkaline solution in batches and then cooled in an ice bath. This “stirring and cooling” process continued until it became nearly transparent. Then, it was diluted to 1500 mL with deionized water, stirring slowly. The supernatant was discarded. The remaining solution was adjusted to neutral by adding a specific amount of 6 mol/L HCl to obtain a well-dispersed cellulose suspension. It was poured into a mold under suction and filtered to form a cylinder, frozen in liquid nitrogen for 5 min, and then transferred to the vacuum chamber of a freeze dryer for 48 h to obtain cellulose aerogel (denoted as CC-CA).

The synthesis of the Fe_3_O_4_-Cu/CA catalyst was carried out as follows: A total of 2 g ferric nitrate and 1.66 g copper nitrate were dissolved in 50 mL ethanol solution. The CC-CA (about 0.6 g) was immersed in the mixed solution under ultrasonics for 20 min and then dried in a 50 °C vacuum drying oven for 20 min. The dried aerogel with metal nitrates was carbonized in a tubular furnace at 600 °C under N_2_ atmosphere for 2 h with a heating rate of 5 °C/min. After the tubular furnace was completely cooled to room temperature, the catalyst was obtained, denoted as Fe_3_O_4_-Cu/CA. Similarly, the reference catalyst Fe_3_O_4_/CA (ferric nitrate was only used during the impregnation process) and Cu/CA (copper nitrate was used during the impregnation process) were prepared, respectively. The complete preparation process of the catalyst is shown in Figure S1.

### 2.3. Characterization Methods

The morphologies of the samples were characterized by scanning electron microscopy (SEM, JEOL-6701) and transmission electron microscopy (TEM, JEOL 2100F) from JEOL (Tokyo, Japan). The X-ray energy spectrometer (EDS) from Oxford Instruments (Oxford, Britain) attached to the TEM was used to detect the element distribution of the sample. The XRD patterns were performed on DX-2700BH from Haoyuan Instrument (Dandong, Liaoning, China)with Cu K_α_ radiation at 30 kV and 25 mA. The pore structure analysis was characterized by using the JW-BK200C BET analyzer from JWGB Instruments (Beijing, China) via the N_2_ adsorption–desorption method at 77 K. FTIR spectra were recorded in the 4000–400 cm^−1^ region with Nicolet IS10 from Thermo Fisher Scientific (Waltham, MA, USA) at room temperature. The Raman spectra were obtained with a DXR3 micro laser Raman spectrometer from Thermo Fisher Scientific (Waltham, MA, USA). The XPS data were obtained with an ESCALab220i-XL electron spectrometer from VG Scientific (Uppsala, Sweden) by using 300 W Al K_α_ radiation. TG analysis was performed on DTG-60AH from Shimadzu Corporation (Kyoto, Japan) from room temperature to 600 °C at a heating rate of 10 °C/min in air. The metal contents in the catalysts were determined by inductively coupled plasma optical emission spectroscopy (ICPOES730)from Agilent Technologies (Santa Clara, CA, USA). The magnetic properties of the catalyst were evaluated by using a Quantum Design superconducting quantum interference device (SQUID VSM) from Quantum Design (San Diego, CA, USA). The degradation of dyes was monitored by the change in absorbance at the maximum absorption wavelength using a UV–visible spectrophotometer (N5000 Plus) from Youke Instrument (Shanghai, China). An EPR test was carried out on Bruker A300-10/12 from Bruker Corporation (Billerica, MA, USA).

### 2.4. Degradation of Organic Pollutants

The heterogeneous Fenton-like catalytic oxidative reaction was evaluated by the degradation of RhB. A typical catalytic cycle was performed in a customized reactor containing 100 mL of 600 ppm RhB and 0.1 g/L catalyst (~10 mg). The initial pH value of the solution was adjusted by dropping the HCl solution under vigorous magnetic stirring in a water bath at a specific temperature for 30 min to establish adsorption–desorption equilibrium. Subsequently, the Fenton reaction was initiated by adding H_2_O_2_ (1 mL, 30 wt%) into the reactor. A quantitative solution (~0.8 mL) was drawn with a sampler at certain time intervals, and the supernatant solution was collected through a 0.22 μm polyphenol ether membrane filter for the following UV–Vis measurement: In detail, the residual catalyst was separated by applying a magnetic field. The UV–Vis absorption at 554.5 nm was recorded to identify the concentration of residual RhB in the reaction system. The metal ion leaching was also measured using ICP-OES. Meanwhile, the recovered catalyst was washed and purified with deionized water for cyclic use.

## 3. Results and Discussions

### 3.1. Characterization

#### 3.1.1. Morphologies, Textural Properties, and Element Compositions

**Figure 1:**
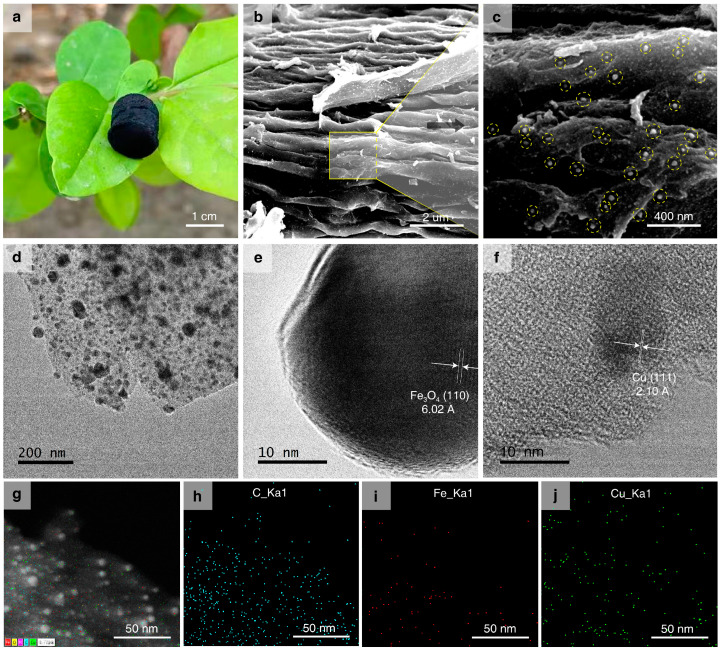
(**a**) Photographic image of the cylindrical catalyst (Fe_3_O_4_-Cu/CA) standing on a leaf. (**b**,**c**) SEM images of the catalyst surface morphology. (**d**–**f**) HRTEM images of the catalyst and its metallic crystal grains. (**g**–**j**) EDS pattern images of the catalyst and its elemental compositions of C, Fe, and Cu species.

The corn-cob-derived carbon aerogel loaded with metal nanoparticles (M/CA) is obtained via the process mentioned above in the Materials and Methods section. The soft, ultralight, and black carbonaceous aerogel is placed on a leaf without significantly bending. It has an ultralight mass density of ~52.2 mg/cm^3^ (Figure 1a). The surface morphology, element composition, and distribution patterns of Fe_3_O_4_-Cu/CA are characterized by using SEM and TEM technology, accompanied by EDS analysis. The SEM and TEM images of the two reference catalysts (Fe_3_O_4_/CA and Cu/CA) are shown in Figure S2. The SEM images of Fe_3_O_4_-Cu/CA show its highly fluffy and complex three-dimensional microstructure (Figure 1b). The soft tissue of the biomass is converted into porous carbon under high-temperature pyrolysis under a N_2_ atmosphere. According to its enlarged SEM image (Figure 1c), a large quantity of nanoparticles is loaded on its surface nanofibers and porous structure. Furthermore, the TEM image shows that the nanoparticles (black dots) are also uniformly dispersed in the bulk structure (Figure 1d). In to the high-resolution TEM (HRTEM) images we observe two distinct crystal particles with different lattice fringe characteristics. The lattice spacing between the (110) planes is ~6.02 nm, which is assigned to the Fe_3_O_4_ crystal shown in Figure 1e. The lattice spacing between the (111) planes is ~2.10 nm, which is also associated with the Cu crystal (Figure 1f). Although these results indicate that Fe probably exists in the form of Fe_3_O_4_, and copper exists in the form of Cu crystals, more evidence is also needed. The EDS pattern images (Figure 1g–j) show the randomly distributed Fe and Cu sites on the surface of the carbonaceous structures in the Fe_3_O_4_-Cu/CA nanocomposites, which also revealed that the reasonable atom ratios of C, O, N, Fe, and Cu are 70.78 wt%, 18.6 wt%, 3.0 wt%, 2.37 wt%, and 5.25 wt%, respectively.

#### 3.1.2. Porous Structures, Crystal Patterns, and Magnetic Properties

**Figure 2:**
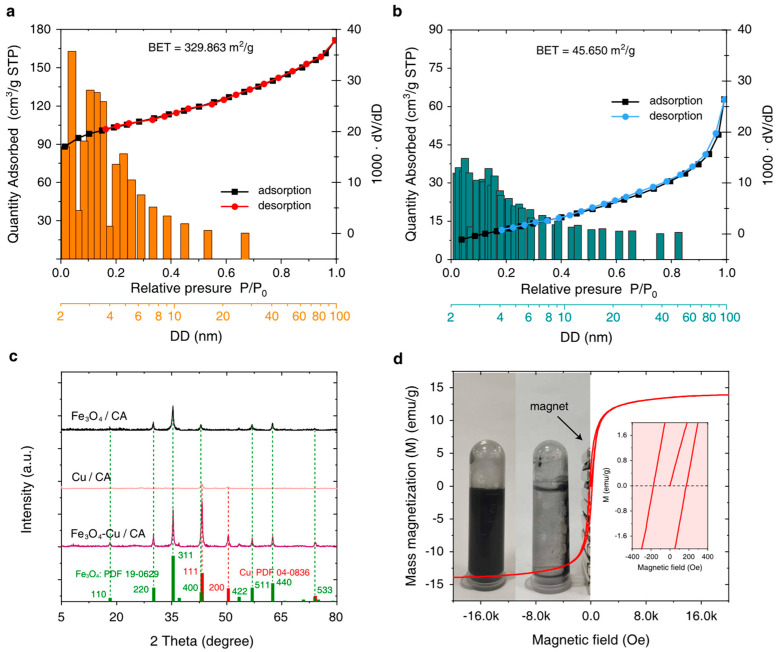
The N_2_ adsorption–desorption isotherms and the pore size distribution bar charts of (**a**) CA and (**b**) Fe_3_O_4_-Cu/CA. (**c**) The XRD patterns of Fe_3_O_4_/CA, Cu/CA, and Fe_3_O_4_-Cu/CA are accompanied by the standard PDF cards of magnetite and copper crystal. (**d**) The VSM curve of Fe_3_O_4_-Cu/CA and the inset image demonstrating its magnetic separation performance.

According to the BET and BJH experimental results shown in Table 1, the BET surface areas of corn-cob-derived CA and Fe_3_O_4_-Cu/CA are 329.86 and 45.65 m^2^/g, respectively. The pore volume of Fe_3_O_4_-Cu/CA is 0.097 cm^3^/g, which is much less than that of CA (0.265 cm^3^/g). The CC-CA is treated as the catalyst support precursor. Interestingly, the mesopore volume almost contributes to the total pore volume in the Fe_3_O_4_-Cu/CA sample, whereas the mesopore volume is 0.15 cm^3^/g in CA. On the other hand, the quantity of micropores in Fe_3_O_4_-Cu/CA is much less than that found in CA, indicating that the metallic ions probably transferred into the micropores when the Fe(NO_3_)_3_-Cu(NO_3_)_2_ ethanol solution diffused into the CC-CA (Figure 2a,b).

The Fe_3_O_4_-Cu/CA catalyst is also characterized by XRD technology with a scan range from 5° to 80° at a rate of 5°/min. The XRD patterns are shown in Figure 2c. By comparing with the standard PDF card, it is inferred that the XRD diffraction peaks of Fe_3_O_4_-Cu/CA are consistent with the characteristic diffraction peaks of Fe_3_O_4_ (PDF 19-0629) and Cu (PDF 04-0836). According to the XRD pattern, the (111), (220), (311), (400), (422), (511), (440), and (533) planes of cubic Fe_3_O_4_ correspond to 2θ degrees of 18.23°, 30.03°, 35.39°, 43.05°, 53.40°, 56.93°, 62.52°, 74.03°, respectively. Furthermore, the diffraction peaks at 43.28° and 50.37° are attributed to the (111) and (200) planes of the cubic Cu crystal. The Fe_3_O_4_ and Cu(0) nanocrystals are believed to form during 600-degree calcination under a N_2_ atmosphere, which also probably generates in situ in the microporous structure of CA.

The magnetic properties of Fe_3_O_4_-Cu/CA have also been investigated by using a vibrating sample magnetometer (VSM) at room temperature, giving its magnetic hysteresis loop, as shown in Figure 2d. It shows that the saturation magnetization of Fe_3_O_4_-Cu/CA is around 13.9 emu/g, with an ultralow coercive magnetic force of nearly 174.2 Oe, indicating its paramagnetism. The Fe_3_O_4_-Cu/CA composite can be easily attracted by a magnet and removed from water within a few minutes, which is favorable for magnetically separating and recovering the catalyst from a Fenton-like reaction system. Therefore, it can be a practically reusable Fenton-like catalyst for water treatment.

#### 3.1.3. Thermogravimetric Analysis

**Figure 3:**
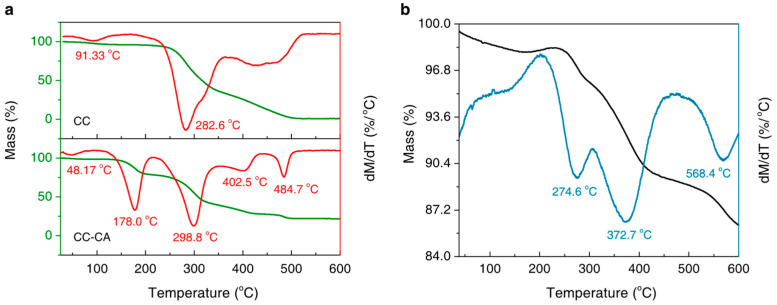
(**a**) TG and DTG curves of the CC and CC-CA; (**b**) TG and DTG results of Fe_3_O_4_-Cu/CA. The heating process is performed from room temperature to 600 °C at a rate of 10 °C/min in air.

Figure 3a shows the TG and DTG curves of the CC and CC-CA. There are three major mass loss stages during the calcination of CC in air, which correspond to the evaporation of absorbed water (91.33 °C), the decomposition of cellulose (282.6 °C), and lignin (its DTG peak ranges from 369.2 °C to 524.6 °C) in sequence. As for the CC-CA, there are five mass loss stages corresponding to the following processes: Firstly, the evaporation of absorbed water mainly happens at 48.17 °C. Secondly, the melting, evaporation, and decomposition of residual urea mainly happen at 178.0 °C. Meanwhile, decomposition products like ammonia and cyanic acid evolve from melting and then react with intact urea to produce biuret. The decomposition of biuret and the formation of cyanuric acid and ammelide also begin in an early temperature range from 230 °C to 360 °C. Thirdly, the decomposition of cellulose mainly happens at 298.8 °C, according to our previous work [24]. Fourthly, the mass loss at 402.5 °C seems to be slight, due to the residual cyanuric acid decomposing to produce ammelide, ammeline, or even melamine gradually. Finally, the minor mass loss at 484.7 °C is likely due to the complete elimination of these generated products.

According to the TG and DTG results of Fe_3_O_4_-Cu/CA shown in Figure 3b, there are three major mass loss stages, except for its dehydration, partial oxidation, and degradation processes in an air atmosphere below 200 °C. The first and second mass loss stages occur mainly at 274.6 °C and 372.7 °C, indicating the degradation process of the carbonized lignocellulose structures. The final mass loss stage happens at 568.4 °C, which is likely due to the decomposition of carbon.

Due to the calcination process of the CC-CA under a N_2_ atmosphere (it is impregnated in advance with metallic nitrate ethanol solution), the carbonaceous structure converts to graphite-like carbon, which also provides a reducing atmosphere at a high temperature for generating Fe(II) and Cu(0) species from their higher-valence states, including Fe(III) and Cu(II). The contents of Fe and Cu in Fe_3_O_4_-Cu/CA are determined by ICP-AES to be 12.34 wt% and 16.51 wt%, respectively.

#### 3.1.4. Surface Chemistry and Carbonized Structures

**Figure 4:**
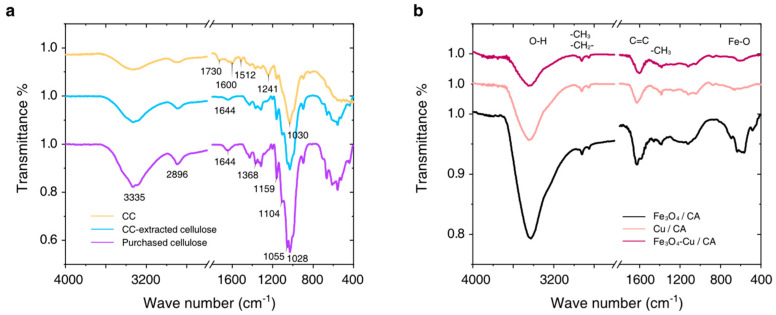
(**a**) FTIR spectra of the CC, CC-extracted cellulose, and purchased cellulose. (**b**) FTIR spectra of Fe_3_O_4_/CA, Cu/CA, and Fe_3_O_4_-Cu/CA.

The FT-IR spectra of the CC, CC-extracted cellulose, and purchased cellulose are shown in Figure 4a. The broad absorption bands at ~3335 cm^−1^ are mainly ascribed to the O–H stretching vibrations of the cellulose, hemicellulose, and pectin. The absorption bands observed at 2896 cm^−1^ and 1368 cm^−1^ can be attributed to the C–H stretching and bending vibrations in methyl, methylene, and methoxy groups for these samples. The absorption band at ~1730 cm^−1^ is significant in the CC IR spectrum, but it almost disappears in the IR spectrum of the CC-extracted cellulose or purchased cellulose. It is ascribed to the stretching vibration of C=O from non-ionic carboxyl groups (-COOH, -COOCH_3_) of pectin in biomass. The absorption band at 1600 cm^−1^ can be assigned to the C=C bond, which is mainly from the benzene ring structure of lignin in the CC IR spectrum, whereas this absorption band shifts to 1644 cm^−1^ due to depolymerization of lignin under cellulose purification. Additionally, the absorption band at 1512 cm^−1^ corresponds to the carbon skeleton vibration mode of the aromatic structure in lignin, which disappears in both the CC-extracted cellulose and the purchased cellulose IR spectra. The absorption band at 1241 cm^−1^ indicates the aromatic oxide (C-O-C stretching vibration mode) from lignin in the CC, which also disappears in the spectra of extracted cellulose and purchased cellulose. On the other hand, the absorption band at 1159 cm^−1^ becomes stronger, which represents the glycosidic bond (C-O-C stretching vibration mode) mainly existing in cellulose. In addition, the absorption bands at 1104 cm^−1^ and 1055 cm^−1^ are obvious, corresponding to the C–O stretching vibration in primary alcohol and secondary alcohol, respectively. The 1030 cm^−1^ IR absorption band indicates the pyran ring skeleton vibration mode. According to these experimental results, the CC-extracted cellulose is very similar to the purchased cellulose in our experiments.

In Figure 4b, the IR absorption curves of the prepared catalysts include Fe_3_O_4_/CA, Cu/CA, and Fe_3_O_4_-Cu/CA. There exist several common IR absorption bands, which are assigned to the O-H bond mainly in alcohol (υ:~3430 cm^−1^, δ: ~1403 cm^−1^), the C-O bond in alcohol (δ: ~1120 cm^−1^, δ: ~1041 cm^−1^), the C-H bond (υ_as_: ~2922 cm^−1^, υ_s_: ~2850 cm^−1^, δ_as_: ~1460 cm^−1^, δ_s_: ~1385 cm^−1^), and the C=C bond in the aromatic structure (υ: 1629~1601 cm^−1^) [25]. The IR absorption band at 586 cm^−1^ is assigned to the Fe-O group [26], which also indicates the existence of Fe oxide in Fe_3_O_4_/CA and Fe_3_O_4_-Cu/CA.

The Raman spectrum of Fe_3_O_4_-Cu/CA (Figure S3) reveals the polarizable vibrations of C=C in aromatics and molecular backbones of carbonaceous materials. Two broad peaks at 1588 cm^−1^ and 1338 cm^−1^ can be observed in the Raman spectrum. The fundamental vibration of the *E*2g stretching modes of all pairs of sp^2^ carbon atoms in aromatic rings (G band) and symmetry breaking at the edges of graphite planes in sp^2^ carbon (D band) are usually significant in the graphite-structured Raman spectrum. Due to the massive, disordered structures of the sample, it has a broad Raman spectrum because of the multiple overlapping peaks of the neighboring carbonaceous species associated with cellulose- and lignin-derived carbon structures. It can be deconvoluted into several bands according to the other reported band assignment methods, which indicates the complex structures of abundant fused aromatic rings with substituent groups [24,27].

#### 3.1.5. Chemical Valence States of Fundamental Elements

**Figure 5:**
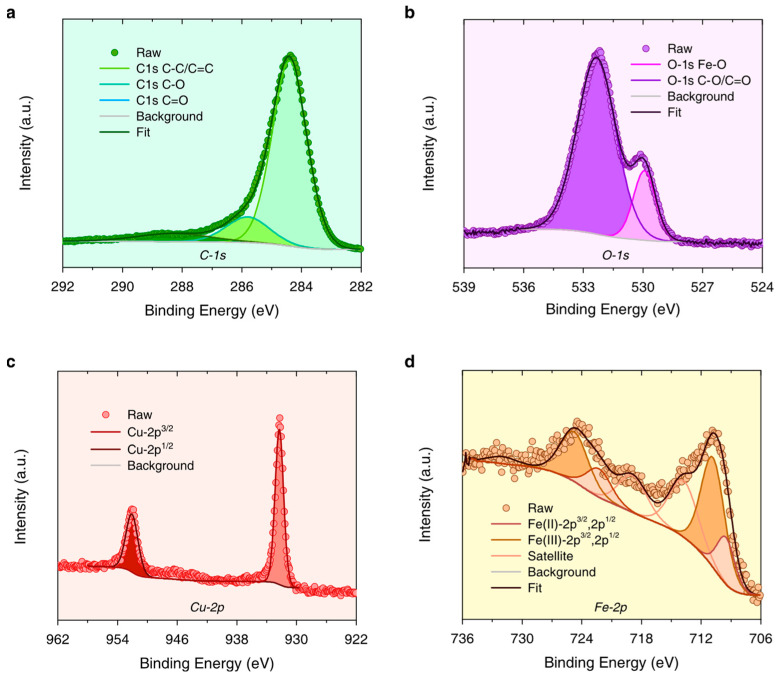
The high-resolution XPS spectra of (**a**) C-1s, (**b**) O-1s, (**c**) Cu-2p, and (**d**) Fe-2p in Fe_3_O_4_-Cu/CA.

In its wide-scan XPS spectrum (Figure S4), Fe_3_O_4_-Cu/CA mainly consists of C, O, Cu, Fe, and other minor impurity elements like Si and Al. According to Figure 5a, the C-1s peak can perform deconvolution into three overlapped peaks, such as peak_1 at 284.4 eV, peak_2 at 285.8 eV, and peak_3, at 288.3 eV. To be specific, C-1s peak_1 is assigned to both C–C and C=C bonds, which is the fundamental linkage bonding of the conjugated carbonized structure in CA. C-1s peak_2 is assigned to C–O bonds derived from the residual lignocellulose structure. C-1s peak_3 is assigned to C=O bonds, which are mainly formed during the pyrolysis process. Otherwise, the O-1s peak can also perform deconvolution into two overlapped peaks, such as peak_1 at 529.9 eV, which is assigned to the O-Fe bonds from Fe oxides, and peak_2 at 532.3 eV, which is assigned to the C–O/C=O bonds (Figure 5b).

Concerning the element of Cu, according to its high-resolution XPS spectrum of Cu-2p (Figure 5c), it can be deconvoluted to the following two peaks: the Cu^(0)^-2p^3/2^ peak at 932.3 eV and the Cu^(0)^-2p^1/2^ peak at 952.1 eV. It is concluded that the Cu element exists in a zero-valence state. As for the Fe element, the Fe-2p peak can be deconvoluted to seven peaks (Figure 5d), and four of them are attributed to Fe^(II)^-2p^3/2^ (709.5 eV), Fe^(III)^-2p^3/2^ (710.8 eV), Fe^(II)^-2p^1/2^ (722.3 eV), and Fe^(III)^-2p^1/2^ (724.8 eV). The other three peaks are assigned to the satellite peaks of Fe^(II)^ or Fe^(III)^. This indicates that the Fe_3_O_4_-Cu/CA catalyst possesses Fe(II) and Fe(III) species from magnetite, which is also proved by its XRD results.

### 3.2. Characterization of Fe_3_O_4_-Cu/CA Catalytic Performance on Fenton-like Degradation of RhB

The Fenton-like catalytic performance of Fe_3_O_4_-Cu/CA is characterized in a well-designed customized reactor (Figure S5a) for the catalytic degradation of high-concentration RhB under different reaction conditions. Several Fenton-like oxidative reaction systems should be characterized, as follows: (1) only with Fe_3_O_4_-Cu/CA; (2) only with H_2_O_2_; (3) with Fe_3_O_4_-Cu/CA and H_2_O_2_; (4) with Fe_3_O_4_/CA and H_2_O_2_; and (5) with Cu/CA and H_2_O_2_. As shown in Figure S5b, in the presence of either only the Fe_3_O_4_-Cu/CA catalyst or only H_2_O_2_ in the reaction system, the RhB degradation could rarely happen (its degradation ratio is less than 3% at 313 K within 60 min reaction time). To be specific, when Fe_3_O_4_-Cu/CA and H_2_O_2_ are simultaneously added, the RhB (at a high initial concentration of 600 ppm) degradation ratio reaches up to nearly 99.1% within 60 min, and the aqueous solution in the reactor becomes nearly colorless at last. This proves that the Fe_3_O_4_-Cu/CA catalyst plays an important role in this Fenton-like reaction process. For either Fe_3_O_4_/CA or Cu/CA, the experimental results show a catalytic degradation ratioless than 5.2% for RhB removal (at an initial concentration of 600 ppm) within 60 min. In general, Fe_3_O_4_-Cu/CA shows more efficient catalytic activity in this reaction (Figure S5c). The kinetic results for the Fenton-like reaction of RhB removal are well matched by the pseudo-first-order (PFO) kinetic model fitting (Figure S6). The observed reaction rate constant *k* is 0.07901 min^−1^, according to the simulated slope of the linear ln(*C*_t_/*C*_0_) versus reaction time (*t*).

**Figure 6:**
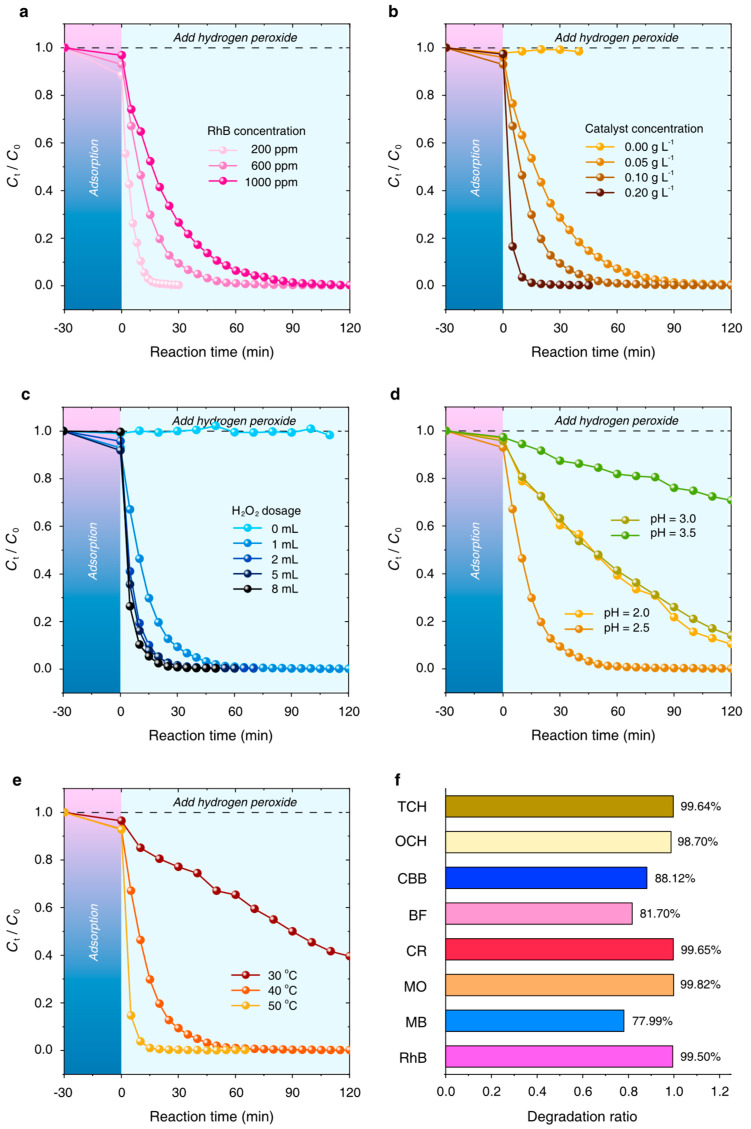
Fenton-like reaction of degrading RhB catalyzed by Fe_3_O_4_-Cu/CA under different reaction conditions: (**a**) different RhB initial concentrations; (**b**) different catalyst dosages; (**c**) different H_2_O_2_ dosages; (**d**) different pH values; and (**e**) different reaction temperatures. In general, all of these reactions follow these conditions: 0.1 g/L catalyst for degradation of 100 mL RhB solution (600 ppm) with 30 wt% H_2_O_2_ (1 mL) at pH 2.5 and 313 K. (**f**) Specified organic pollutants, including 8 kinds, in a volume of 20 mL with an initial concentration of 100 ppm, simultaneously adding 5 mg of catalyst, 200 μL of H_2_O_2_ (30 wt%), at pH = 2.5, 313 K, within a reaction time of 1 h.

The initial RhB concentration is usually considered one of the most critical factors that determine its degradation rate dramatically. To elucidate the importance of initial dye concentration, 100 mL of 200 ppm, 600 ppm, and 1000 ppm of RhB solutions are investigated in a Fenton-like oxidative reaction system, as shown in Figure 6a. The degradation rate of RhB decreases as the initial RhB concentration increases. Within almost 60 min, RhB removal is promoted by the Fe_3_O_4_-Cu/CA catalyst, and the residual percentage of RhB decreases to 0.1% (200 ppm), 0.9% (600 ppm), and 6.3% (1000 ppm), respectively. The results indicate that the amount of ·OH generated by the Fe_3_O_4_-Cu/CA/H_2_O_2_ Fenton-like reaction in this condition is sufficient for the complete degradation of RhB with a high initial concentration up to 1000 ppm.

The factor of catalyst dosage is also investigated in Figure 6b. It indicates that the degradation ratio of RhB increased from 71.4% to 99.7% with the increase in catalyst dosage from 0.05 mg/L to 0.2 mg/L. A total 90.6% of RhB can be removed in this Fenton-like oxidative reaction system when the catalyst dosage is 0.1 g/L, which is considered the optimum condition in the experiments.

The effect of the H_2_O_2_ dosage is characterized by varying the H_2_O_2_ solution volume, being 1 mL, 2 mL, 5 mL, and 8 mL. In Figure 6c, the degradation ratio of RhB monotonically increases from 91.6% (1 mL H_2_O_2_ solution) to 99.3% (8 mL H_2_O_2_ solution) within 30 min. Considering the atomic economy, we decided that the H_2_O_2_ dosage should be 1 mL in the experiments.

The effect of the initial pH value on the degradation rate of RhB (600 ppm) catalyzed by the Fe_3_O_4_-Cu/CA catalyst is shown in Figure 6d. It is observed that the degradation rate of RhB increases as the pH value ranges from 2.0 to 2.5 and then decreases with a pH value ranging from 2.5 to 3.5. To be specific, it reveals that a certain initial pH value of 2.5 is favorable for obtaining high catalytic efficiency of Fe_3_O_4_-Cu/CA in promoting the RhB removal by a Fenton-like reaction. The results are likely attributed to the appropriate Fe leaching at pH = 2.5, which favors Fe(II)/H_2_O_2_ reaction to produce ·OH. Moreover, the precipitation of more iron hydroxides/oxides could cover the catalyst surface and hinder the reaction rate of producing ·OH. The optimum pH is considered to be 2.5 for the Fe_3_O_4_-Cu/CA catalyst in the heterogeneous Fenton-like degradation of high-concentration dye solutions.

Figure 6e shows the degradation rate of RhB at different temperatures. The residual RhB ratio (*C*_t_/*C*_0_) decreases from 77.1% to 0.2% within 30 min as the temperature increases from 303 K to 323 K. It has been proven that the enthalpy of the RhB oxidative degradation is positive, which indicates that this process is endothermic. However, higher temperatures require more energy consumption. Therefore, the applicable temperature is considered to be 313 K in the experiments.

To evaluate the Fenton-like oxidative reaction for other organic pollutants, which is catalyzed by Fe_3_O_4_-Cu/CA, the corresponding experiments to identify its catalytic performance are performed as shown in Figure 6f. All eight kinds of organic pollutants, such as tetracycline hydrochloride (TCH), oxytetracycline hydrochloride (OCH), coomassie brilliant blue G250 (CBB), basic fuchsin (BF), congo red (CR), methylene orange (MO), methylene blue (MB), and rhodamine B (RhB), can be removed to a great extent, and the degradation ratio of each substrate remains more than 78%. Additionally, the catalytic performance comparison between Fe_3_O_4_-Cu/CA and other reported catalysts is summarized in Table 2. In these reaction systems, RhB removal quantity, when using micromolar units (μmol), can be considered as a key index to identify the catalytic activity of different catalysts. In this work, the RhB degradation reaction requires much less catalyst and H_2_O_2_ dosage. More than 100 μmol RhB is completely degraded at a pH of 3.0 within 60 min in the Fenton-like reaction. All of these results indicate that Fe_3_O_4_-Cu/CA has great application prospects and good universality for the application of ultrahigh-concentration organic pollutant removal at ultralow cost in wastewater treatment.

### 3.3. Reusability and Catalytic Stability

To evaluate the reusability and stability of Fe_3_O_4_-Cu/CA, cycle catalytic experiments of RhB removal are performed in 10 consecutive runs. The degradation ratio of RhB is measured within 10, 20, and 30 min in each cycle. As each cycle ends, the used catalyst is suction-filtered and separated from the above aqueous solution for the next run.

**Figure 7:**
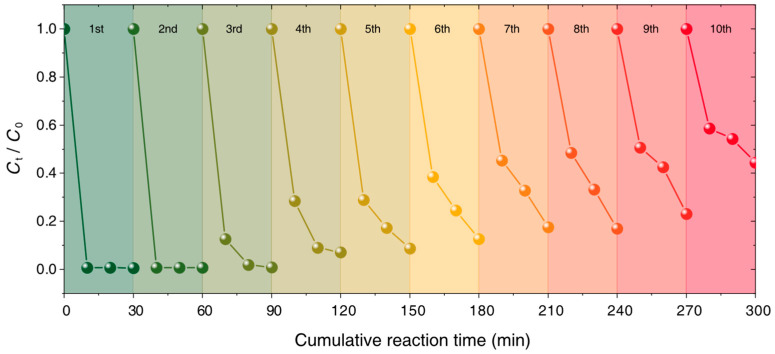
Fenton-like oxidative degradation ratio of RhB as the reaction time operates within 10 cycles. Reaction condition: 15 mg Fe_3_O_4_-Cu/CA for catalytic degradation of 20 mL RhB solution (100 ppm) with 200 μL dosage of H_2_O_2_ (30 wt%) in conditions of pH = 2.5 and 313 K.

The concentration of leached Fe and Cu ions from Fe_3_O_4_-Cu/CA at pH = 2.5 during a Fenton-like reaction cycle is measured using the ICP-OES method. The total metal leaching observed only accounted for 1.4% of Fe and 4.0% of Cu from the total metal content (15 mg catalyst dosage, 20 mL RhB solution (600 ppm), 200 μL 30 wt% H_2_O_2_ dosage, at pH of 2.5, at 40 °C), revealing that the metal leaching will not cause secondary contaminations to the treated water. As depicted in Figure 7, the degradation ratio of RhB is still more than 80% in the 8th cycle, which indicates the good reusability and stability of the Fe_3_O_4_-Cu/CA catalyst for the Fenton-like oxidative reaction of RhB removal. According to the TEM images of the fresh and used Fe_3_O_4_-Cu/CA in Figure S7, there is no significant change in their structures or morphologies before and after use.

### 3.4. Catalytic Oxidation Mechanism of Fe_3_O_4_-Cu/CA in Fenton-like System

#### 3.4.1. Influence of Co-Existing Ions

There are various anions in the actual sewage system, which may have great or minor influences on the degradation efficiency of organic pollutants in the Fenton-like oxidative reaction system [36,37]. In order to answer this question, four kinds of fundamental anions are individually added to the Fenton-like oxidative system in the form of 5 mM sodium salt. In the 100 mL of 600 ppm RhB solution with 1 mL dosage of 30 wt% H_2_O_2_ at 313 K, the initial pH value is adjusted to 2.5. First of all, 5 mM of NaCl, NaNO_3_, and Na_2_SO_4_ are individually added to the reaction system without a significant impact on the pH value. As shown in Figure S8, the influence of ${Cl}^{-}$ or ${NO}_{3}^{-}$ on the Fenton-like oxidative system is negligible. However, the degradation ratio of RhB dropped down to 66% in the presence of ${SO}_{4}^{2-}$, indicating that there is a critical mechanism contributing to the negative effect on the catalytic process. This is probably ascribed to the formation of persulfate, ${S_{2}O}_{8}^{2-}$, which may consume the oxidative species generated from H_2_O_2_, thus depleting the amount of ·OH in the system.

On the other hand, the addition of 5 mM Na_2_CO_3_ has a great impact on the initial pH value of the reaction system. As a result, the solution’s pH increases from 2.5 to 9.7 in this case, which may precipitate the leached Fe^3+^/Fe^2+^ and Cu^2+^/Cu^+^ in the Fenton-like catalytic reaction; moreover, the RhB degradation ratio dramatically decreases to 12% within 30 min. It is notable that the oxidation potential of ·OH also decreases significantly as the pH value rises, which may also result in an obvious decrease in oxidation efficiency.

#### 3.4.2. Active Oxidative Species in Fe_3_O_4_-Cu/CA Catalytic Fenton-like Reaction System

To investigate the major active oxidative species generated from H_2_O_2_, which is catalyzed by Fe_3_O_4_-Cu/CA, reactive oxygen species (ROS) quenching tests are carried out. Moreover, the corresponding electron paramagnetic resonance (EPR) spectra of DMPO- and TEMP-adducts are also measured as supplementary evidence.

**Figure 8:**
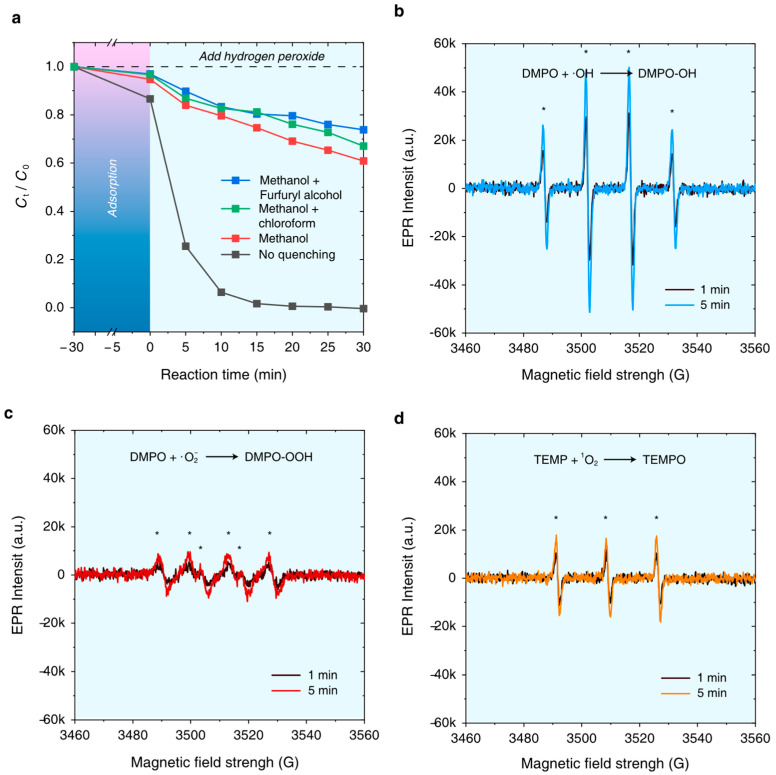
(**a**) Influences of different radical scavengers on the degradation ratio of RhB in a Fenton-like oxidative reaction system catalyzed by Fe_3_O_4_-Cu/CA (experimental conditions: 0.1 g/L Fe_3_O_4_-Cu/CA catalyst, 100 mL RhB (600 ppm) solution, 1 mL dosage of 30 wt% H_2_O_2_ at 313 K with initial pH = 2.5). The EPR spectra of (**b**) DMPO-OH adducts formed by capturing ·OH, (**c**) DMPO-OOH adducts formed by capturing ·O_2_^−^, and (**d**) TEMPO adducts formed by capturing ^1^O_2_ in a Fenton-like oxidative reaction system catalyzed by Fe_3_O_4_-Cu/CA.

As shown in Figure 8a, the degradation ratio of RhB is inhibited to some extent after the addition of a quenching agent [21]. It decreases from 99.7% to 39.1% while adding methanol in this Fenton-like oxidative reaction, indicating that ·OH plays a dominating role in the degradation of RhB. Furfuryl alcohol (FFA) could quench ^1^O_2_, and it also shows high reactivity towards ·OH. Therefore, FFA and methanol are simultaneously added to the system, and the degradation ratio of RhB even further decreases to 26.1%, which proves that ^1^O_2_ also contributes to the oxidative process in the system. Otherwise, chloroform is commonly used as the quenching agent of ·O_2_^−^. The degradation ratio of RhB decreases to 33.0% with the addition of both methanol and chloroform. It is concluded that ·OH is the primary active oxygen species in the Fe_3_O_4_-Cu/CA-catalyzed Fenton-like oxidative reaction system.

In EPR tests, DMPO is used to capture ROS, including ·OH (generating DMPO-OH) and ^1^O_2_ (generating DMPO-OOH), whereas TEMP is used to capture ·O_2_^−^ (generating TEMPO) in the Fenton-like oxidative reaction system. As shown in Figure 8b, the EPR intensity of the quadruplet characteristic peaks within the reaction time of 5 min, which are assigned to the typical signal of DMPO-OH with an intensity ratio of 1:2:2:1, is larger than that of the peaks observed at the beginning (1 min). The EPR peaks are assigned to the existence of DMPO-OOH, as shown in Figure 8c. It becomes slightly larger than that observed at the beginning of the reaction (1 min). Moreover, TEMP is used to capture ·O_2_^−^, and the corresponding signal of TEMPO is observed with a slight increase as the reaction proceeds from 1 min to 5 min (Figure 8d). All of these results follow the conclusions drawn from the radical capture experiments.

#### 3.4.3. Co-Catalytic Mechanism of the Fe_3_O_4_-Cu/CA Fenton-like Oxidative Reaction System

**Figure 9:**
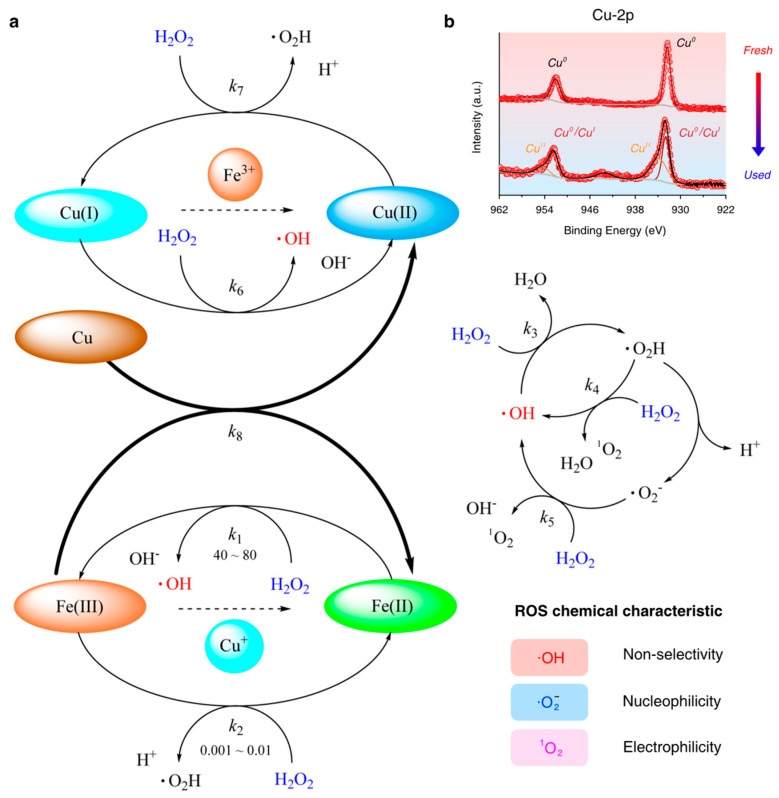
(**a**) A schematic diagram of the co-catalytic mechanism of Fe-Cu sites in this Fenton-like reaction system; and (**b**) the high-resolution XPS spectra of Cu-2p in both fresh and used Fe_3_O_4_-Cu/CA catalysts.

The schematic diagram of the co-catalytic mechanism in this Fenton-like reaction by the Fe-Cu bimetallic catalyst is thoroughly depicted in Figure 9a. To our knowledge, the Fe (II) ion is considered to be the key reactive agent in generating ·OH with a high reaction kinetic value of *k*_1_ in the Fenton system [21]. The Fe(II)/H_2_O_2_ reaction (R. 1) is highly activated in acidic conditions. However, the Fe(III)/H_2_O_2_ reaction (R. 2) is unappreciated in efficiently generating ·OH because of its relatively lower reaction rate (*k*_2_ is much lower than *k*_1_), which is commonly accepted as a linkage in the redox cycle reaction of Fe(III)/Fe(II), and this process is also considered as the rate-limiting process in Fe-based Fenton-like systems. Except for generating ·OH, other ROS (such as ·O_2_^−^ and ^1^O_2_) are mainly produced from reactions R. 3, R. 4, and R. 5 in the system.

All of the above reactions are shown as follows:

(1) $$\equiv Fe\left(II\right)+H_{2}O_{2}\to \equiv Fe(III)+{OH}^{-}+\cdot OH$$

(2) $$\equiv Fe\left(III\right)+H_{2}O_{2}\to \equiv Fe\left(II\right)+\cdot O_{2}H+H^{+}$$

(3) $$\cdot OH+H_{2}O_{2}\to \cdot O_{2}H+H_{2}O$$

(4) ·O2H+H2O2→·OH+O21+H2O

(5) ·O2−+H2O2→OH−+O21+·OH

It is accepted that Fe(III) can be reduced to Fe(II) in the presence of Cu or Cu(I) [29,38]. Otherwise, Fe(III) is easily precipitated by the formation of ferric hydroxide at a pH above 3.0. In the Cu-2p spectrum of the fresh Fe_3_O_4_-Cu/CA catalyst, Cu mainly exists in a zero-valence state, as discussed in the previous section. However, there are additional sub-peaks (Cu^(II)^-2p^3/2^ at 933.8 eV and Cu^(II)^-2p^1/2^ at 954.4 eV) that appear newly in the Cu-2p spectrum of the used catalyst, which are shown in Figure 9b. This indicates that non-zero-valence Cu species are not only generated from the Cu(I)/H_2_O_2_ reaction (R. 6) and Cu(II)/H_2_O_2_ reaction (R. 7), but are also originally produced from the second cyclic conversion between Fe(III) and Fe(II) with the assistance of Cu(0) and Cu(I) species (R. 8 and R. 9) in the Fe_3_O_4_-Cu/CA-catalyzed Fenton-like system [21,39], eventually improving the catalytic properties.

These reactions are also shown as follows:

(6) $$\equiv Cu\left(I\right)+H_{2}O_{2}\to \equiv Cu\left(II\right)+{OH}^{-}+\cdot OH$$

(7) $$\equiv Cu\left(II\right)+H_{2}O_{2}\to \equiv Cu\left(I\right)+\cdot O_{2}H+H^{+}$$

(8) $${Cu+2Fe}^{3+}\to {Cu}^{2+}+2{Fe}^{2+}$$

(9) $${{Cu}^{+}+Fe}^{3+}\to {Cu}^{2+}+{Fe}^{2+}$$

In general, Fe(II) and Cu(I) can promote H_2_O_2_ decomposition to produce ·OH. Otherwise, Cu(0) facilitates the regeneration of Fe(II). In addition, the standard reduction potentials of Cu(II)/Cu(I) and Cu(II)/Cu are 0.153 V and 0.3419 V, respectivley, while that of Fe(III)/Fe(II) is 0.771 V, which benefits the continuous redox cycles of Fe(III)/Fe(II) and Cu(II)/Cu(I) in the Fenton-like system [40].

## 4. Conclusions

The bimetallic catalyst Fe_3_O_4_-Cu/CA is prepared by using biomass corn-cob-derived carbon aerogel as support, which exhibits high catalytic performance and stability for the degradation of RhB at ultrahigh concentrations of up to 1000 ppm and low catalyst and H_2_O_2_ dosage. The degradation ratio of RhB (initial concentration of 1000 ppm for 100 mL volume) is 93.7% within 60 min at pH 2.5, 40 °C, 1 mL 30 wt% H_2_O_2_ dosage, and 0.1 g/L catalyst. The catalyst reveals good catalytic stability and is universally applicable to different organic pollutants. In addition, the cellulose aerogel used in the preparation of Fe_3_O_4_-Cu/CA can be extracted from biomass sources, which are widely available and low-cost. It also provides an in situ reducing atmosphere for generating Fe(II) and Cu(0) species from their higher-valence metal ions, including Fe(III) and Cu(II). The key ROS involve ·OH (mainly), ^1^O_2_, and ·O_2_^−^, according to quenching experiments, which are also identified by ROS-captured products in EPR tests. The synergistic effect between Fe(III)/Fe(II) and Cu/Cu(I)/Cu(II) species played a key role in promoting the reduction of Fe(III) to Fe(II) and enhancing the continuous mass production of ·OH, considered to play a dominating role in the degradation of organic pollutants. All of these results indicate that Fe_3_O_4_-Cu/CA is an attractive low-cost, high-efficiency approach for ultrahigh-concentration organic pollutant degradation in practical wastewater treatment applications.

## Figures and Tables

**Table 1:** Results of the BET and BJH experimental analysis of CC, CA, and Fe_3_O_4_-Cu/CA.

Sample Name	S_BET_ m^2^/g	S_meso_ m^2^/g	V_total_ cm^3^/g	V_meso_ cm^3^/g	Mid-Value D_micro_ nm	Average D_meso_ nm
CC	1.4	-	-	-	-	-
CA	329.9	103.4	0.26	0.15	3.97	5.77
Fe_3_O_4_-Cu/CA	45.6	52.0	0.09	0.10	3.71	7.80

**Table 2:** The catalytic performance comparison between Fe_3_O_4_-Cu/CA and other reported catalysts.

Catalysts	Catalyst C/Dosage (g/L)/(mg)	Initial C_RhB_ (mg/L)	N_RhB_ Removal (μmol)	H_2_O_2_/RhB mol/mol	Initial pH	Reaction Temperature (°C)	Reaction Time (min)	Degradation Ratio	Ref.
Fe_3_O_4_-Cu/CA	0.1/10	1000	195.6	46.9	2.5	40	60	93.7%	This work
Fe_3_O_4_-Cu/CA	0.1/10	600	124.1	78.2	2.5	40	60	99.1%	This work
Fe_3_O_4_-Cu/CA	0.1/10	600	107.7	78.2	3	40	60	86%	This work
CuO–FeSe_2_	0.4/40	1	0.2	-	-	rt, light	180	96%	[12]
FeCu/BC_600-2_	0.2/10	10	1.0	95.8	3	30	60	100%	[22]
zero-valent iron	0.5/250	47.9	48	80	4	25	60	98%	[28]
Cu/Fe-X type zeolite	1.0/100	100	20.9	938	7	60	90	99.9%	[29]
GF/CuS/Fe_3_O_4_	0.1/20	5.8	2.37	4044	-	rt, light	60	98%	[30]
Cu-Fe_3_O_4_/Cu/C	0.1/20	10	4.18	479	3	30	45	100%	[31]
Fe-Cu/γ-Al_2_O_3_	2.2/1100	200	209	739.6	7	70	3.5	99.9%	[32]
α-Fe_2_O_3_/γ-Fe_2_O_3_	0.2/10	8	0.83	10.8	7	rt, light	12	99.2%	[33]
Fe_3_O_4_/CHC	2.4/24	10	0.21	47.9	2	rt	180	98.3%	[34]
Cu/ZnFe_2_O_4_	2.0/20	20	0.41	-	8	rt, light	140	98.1%	[35]

rt: Room temperature; -: no information.

## Data Availability

The datasets generated during and/or analyzed during the current study are available from the corresponding author upon reasonable request.

## Supplementary Materials

The following supporting information can be downloaded at: https://www.mdpi.com/article/10.3390/nano15080614/s1, Figure S1: The preparation process of the Fe-Cu/CA catalyst; Figure S2: The TEM images of the catalyst Fe_3_O_4_/CA (a,b) and Cu/CA (c,d). Figure S3: The Raman spectrum of Fe_3_O_4_-Cu/CA. Figure S4: The wide-scan XPS pattern of (a) Fe_3_O_4_-Cu/CA and (b) its partially enlarged pattern. Figure S5: (a) The photograph of a well-designed customized reactor for performing the Fenton-like reaction of RhB removal to identify the catalytic performance of the prepared catalyst. (b) Fenton-like reaction of degrading RhB catalyzed by Fe_3_O_4_-Cu/CA with or without H_2_O_2_,comparison with thereaction without catalyst only in the presence of H_2_O_2_; (c) Fenton-like reaction of degrading RhB catalyzed by Fe_3_O_4_/CA, or Cu/CA, or Fe_3_O_4_-Cu/CA. Figure S6: The Fenton-like reaction of RhB removal under the catalysis of Fe_3_O_4_-Cu/CA (reaction condition: 40 °C, 100 mL 600-ppm RhB solution, 0.1 g/L catalyst usage, pH = 2.5, 1 mL H_2_O_2_ dosage), which is simulated by the pseudo-first-order (PFO) kinetic model. Figure S7: TEM images of (a,b) the fresh Fe_3_O_4_-Cu/CA catalyst and (c,d) the used Fe_3_O_4_-Cu/CA catalyst. Figure S8: The catalytic Fenton-like oxidative degradation of RhB influenced by different co-existing anions (experimental conditions: 0.1 g/L Fe_3_O_4_-Cu/CA catalyst, 100 mL RhB (600 ppm) solution, 1 mL dosage of 30 wt% H_2_O_2_ at 313 K with initial pH = 2.5). Figure S9: The zeta potential of Fe_3_O_4_-Cu/CA under different pH conditions. Table S1: The Fenton-like reaction of degrading 100 mL of 600 ppm RhB with adding 9.8 mmol of H_2_O_2_, at 40 °C under different conditions.
