# Supplementary material for: Biomass Cellulose-Derived Carbon Aerogel Supported Magnetite-Copper Bimetallic Heterogeneous Fenton-like Catalyst Towards the Boosting Redox Cycle of ≡Fe(III)/≡Fe(II)
Source: Nanomaterials (Basel). 2025 Apr 16;15(8):614. doi: 10.3390/nano15080614 (PMC12029258; doi:10.3390/nano15080614)
Supplement: Supplementary file 1 [file nanomaterials-15-00614-s001.zip › nanomaterials-3539482-supplementary.pdf]

Supporting Information

Biomass-source cellulose-derived carbon aerogel supported magnetite-copper bimetallic heterogeneous Fenton-like catalyst towards the boosting redox cycle of ≡Fe(III)/≡Fe(II)

Qiang Zhao [a, b, *], Jiawei Yang [b], Jiayi Xia [a], Gaotian Zhao [b], Yida Yang [c], Zongwei Zhang [d], Jing Li [c], Fang Wei [a, b, *], Weiguo Song [c]

[a] College of Science, Civil Aviation University of China (CAUC), Tianjin, 300300, China

[b] College of Aerospace Engineering, Civil Aviation University of China (CAUC), Tianjin, 300300, China

[c] Laboratory of Molecular Nanostructure and Nanotechnology, Institute of Chemistry, Chinese Academy of Sciences, Beijing, 100190, China

[d] Science and Technology Innovation Research Institute, Civil Aviation University of China (CAUC), Tianjin, 300300, China

* Corresponding author. College of Science, Civil Aviation University of China (CAUC), Tianjin, 300300, China

Email address: zhao-q@cauc.edu.cn (Q. Zhao)

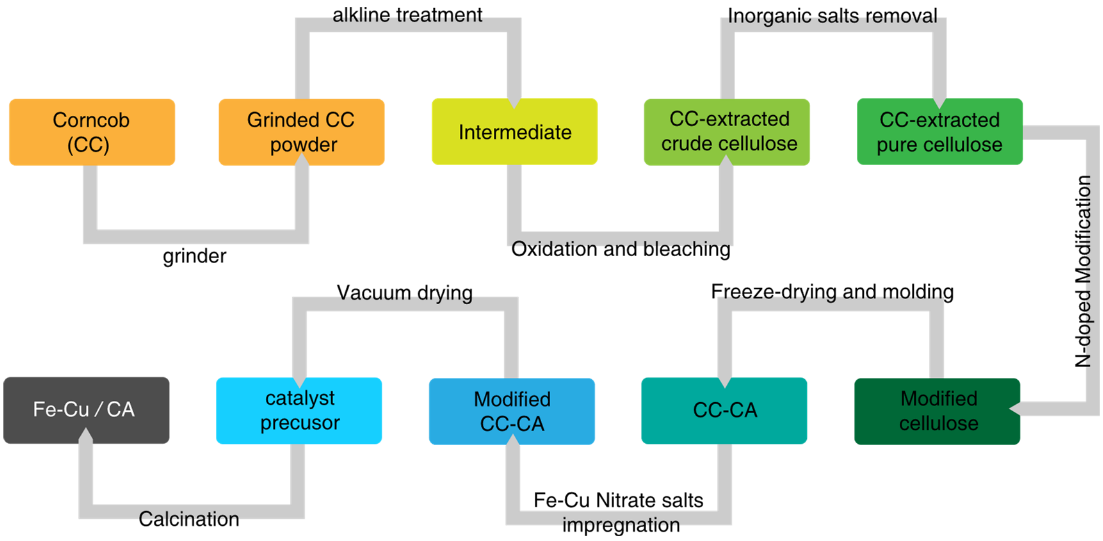

Figure S1 The preparation process of the Fe-Cu/CA catalyst.

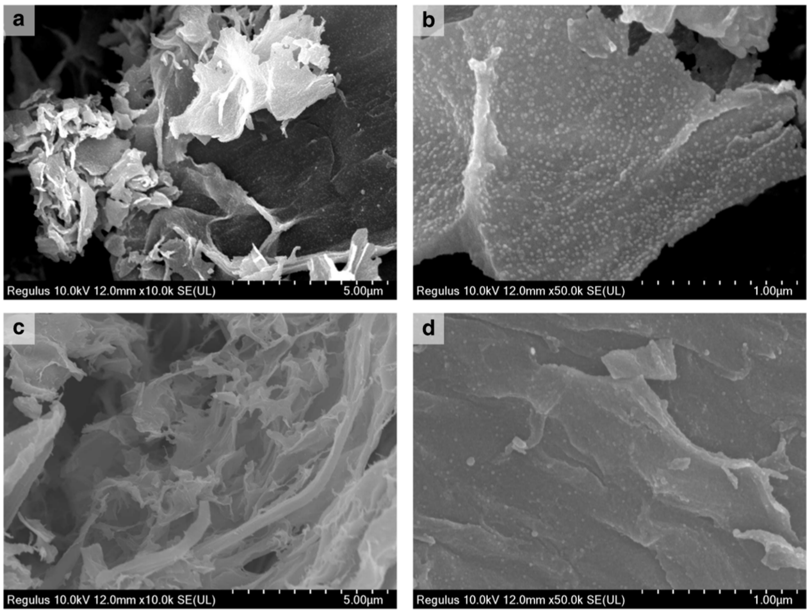

Figure S2 The TEM images of the catalyst Fe_3O_4/CA (a,b) and Cu/CA (c,d).

The Raman spectrum of Fe_3O_4-Cu/CA (**Figure S3**) reveals the polarizable vibrations of C=C in aromatics and molecular backbones of carbonaceous materials. Two broad peaks at 1588 cm^{-1} and 1338 cm^{-1} can be observed in the Raman spectrum. The fundamental vibration of the *E*2g stretching modes of all pairs of sp^2 carbon atoms in aromatic rings (G band) and symmetry breaking at the edges of graphite planes in sp^2 carbon (D band) are usually significant in the graphite-structured Raman spectrum. Due to the massive disordered structures of the sample, it has a broad Raman spectrum because of the multiple overlapping peaks of the neighboring carbonaceous species associated with cellulose- and lignin-derived carbon structures. It also indicates the complex structures of abundant fused aromatic rings with substituent groups.

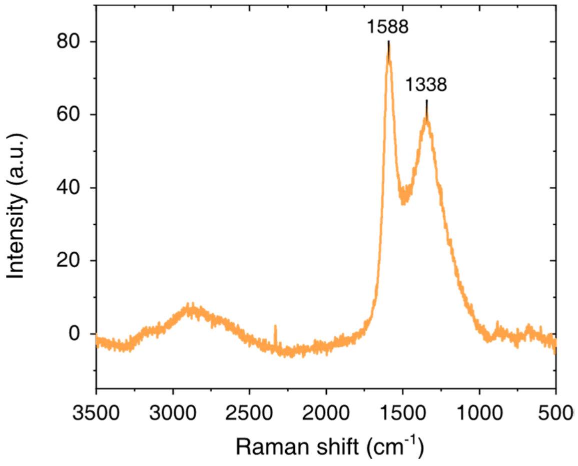

Figure S3 The Raman spectrum of Fe_3O_4-Cu/CA.

In its wide-scan XPS spectrum (**Figure S4**), Fe_3O_4-Cu/CA mainly consists of C, O, Cu, Fe, and other minor impurity elements like Si and Al.

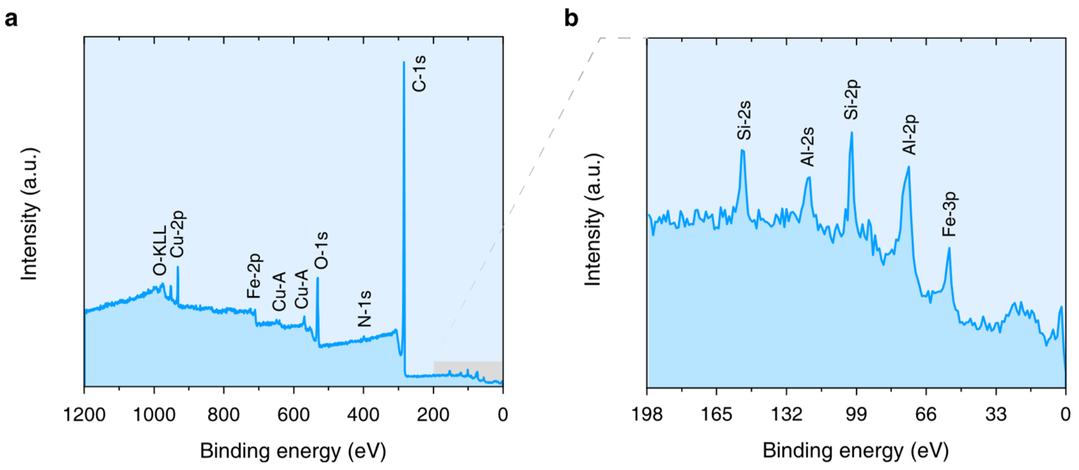

Figure S4 The wide-scan XPS pattern of (a) Fe_3O_4-Cu/CA and (b) its partially enlarged pattern.

The Fenton-like catalytic performance of Fe_3O_4-Cu/CA is characterized in a well-designed customized reactor (**Figure S5a**) for the catalytic degradation of high-concentration RhB under different reaction conditions. Several Fenton-like oxidative reaction systems should be characterized: 1) only with Fe_3O_4-Cu/CA; 2) only with H_2O_2; 3) with Fe_3O_4-Cu/CA and H_2O_2; 4) with Fe_3O_4/CA and H_2O_2; 5) with Cu/CA and H_2O_2. As shown in **Figure S5b**, in the presence of either only Fe_3O_4-Cu/CA catalyst or only H_2O_2 in the reaction system, the RhB degradation could rarely happen (its degradation ratio is less than 3% at 313 K within 60 min reaction time). To be specific, when Fe_3O_4-Cu/CA and H_2O_2 are simultaneously added, the RhB (at a high initial concentration of 600 ppm) degradation ratio reaches up to nearly 99.1% within 60 min, and the aqueous solution in the reactor becomes nearly colorless at last. It proves that the Fe_3O_4-Cu/CA catalyst plays an important role in this Fenton-like reaction process. When compared with Fe_3O_4-Cu/CA, either Fe_3O_4/CA or Cu/CA show merely catalytic degradation ratio that is less than 5.2% for RhB removal (at an initial concentration of 600 ppm) within 60 min, which reveals that Fe_3O_4-Cu/CA is much more efficient than either Fe_3O_4/CA or Cu/CA (**Figure S5c**).

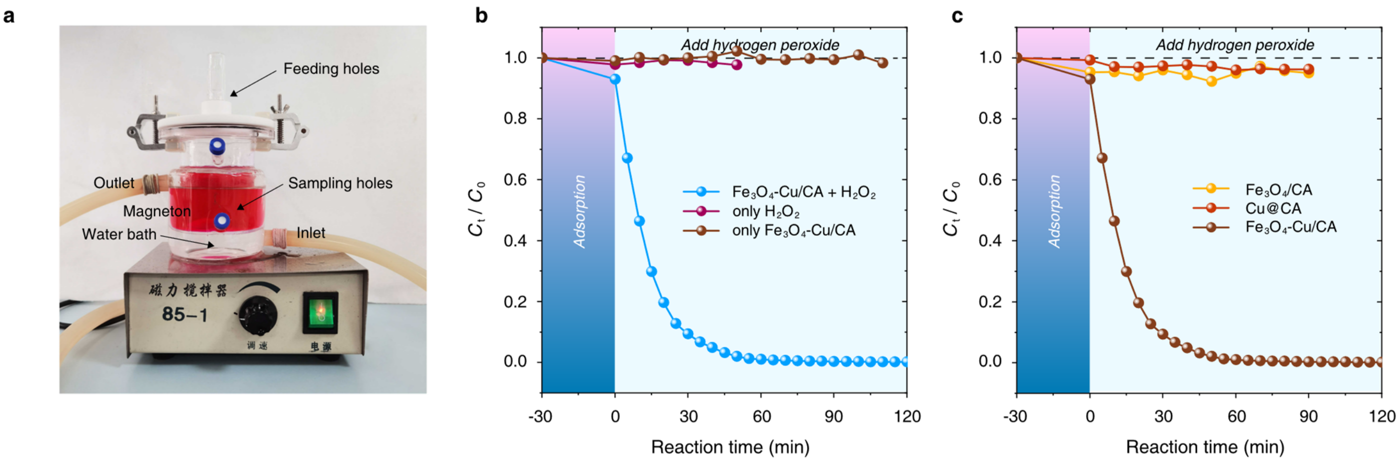

Figure S5 (a) The photograph of a well-designed customized reactor for performing the Fenton-like reaction of

RhB removal to identify the catalytic performance of the prepared catalyst. (b) Fenton-like reaction of degrading RhB catalyzed by Fe_3O_4-Cu/CA with or without H_2O_2, comparison with reaction without catalyst only in the presence of H_2O_2; (c) Fenton-like reaction of degrading RhB catalyzed by Fe_3O_4/CA, or Cu/CA, or Fe_3O_4-Cu/CA.

The Fenton-like degradation rate of RhB followed the pseudo-first-order (PFO) kinetic model (**Figure S6**).

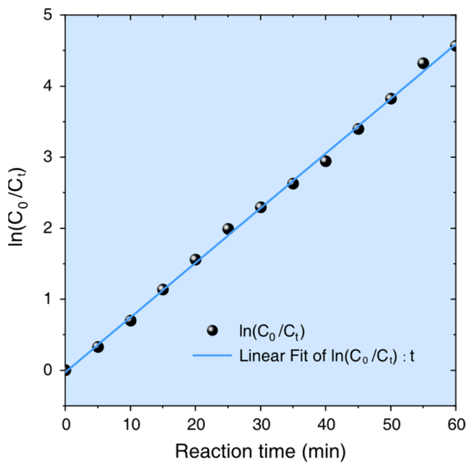

Figure S6 The Fenton-like reaction of RhB removal under the catalysis of Fe_3O_4-Cu/CA (reaction condition: 40 ºC, 100 mL 600-ppm RhB solution, 0.1 g/L catalyst usage, pH = 2.5, 1 mL H_2O_2 dosage), which is simulated by the pseudo-first-order (PFO) kinetic model.

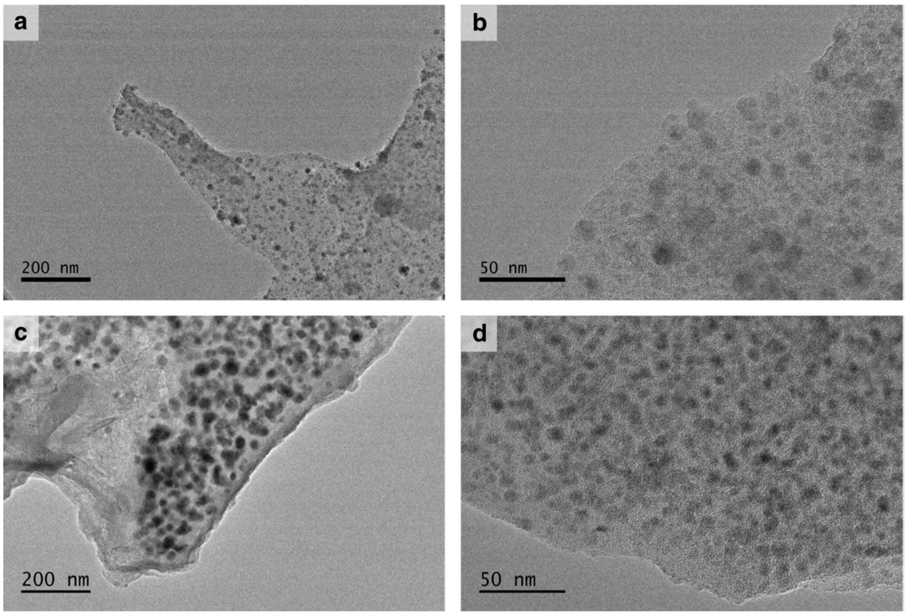

Figure S7 TEM images of (a,b) the fresh Fe3O4-Cu/CA catalyst and (c,d) the used Fe3O4-Cu/CA catalyst.

In order to answer this question, four kinds of fundamental anions are individually added to the Fenton-like oxidative system in the form of 5 mM sodium salt. In the 100 mL of 600 ppm RhB solution with 1 mL dosage of 30 wt% H_2O_2 at 313 K, the initial pH value is adjusted to 2.5. First of all, 5 mM of NaCl, $NaNO_3$, and Na_2SO_4 are individually added to the reaction system without a significant impact on the pH value. As shown in **Figure S8**, the influence of Cl^- or NO_3^- on the Fenton-like oxidative system is negligible. However, the degradation ratio of RhB dropped down to 66% in the presence of SO_4^{2-}, indicating that there is a critical mechanism contributing to the negative effect on the catalytic process. This is probably ascribed to the formation of persulfate, $S_2O_8^{2-}$, which may consume the oxidative species generated from H_2O_2, thus depleting the amount of ·OH in the system.

On the other hand, the addition of 5 mM Na_2CO_3 has a great impact on the initial pH value of the reaction system. As a result, the solution pH increases from 2.5 to 9.7 in this case, which

may precipitate the leached Fe^{3+}/Fe^{2+} and Cu^{2+}/Cu^{+} in the Fenton-like catalytic reaction; the RhB degradation ratio dramatically decreases to 12% within 30 min. It is notable that the oxidation potential of ·OH also decreases significantly as the pH value rises, which may also result in an obvious decrease in oxidation efficiency.

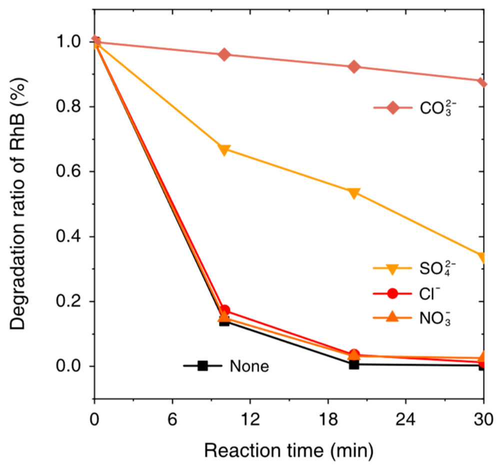

Figure S8 The catalytic Fenton-like oxidative degradation of RhB influenced by different co-existing anions (experimental conditions: 0.1 g/L Fe_3O_4-Cu/CA catalyst, 100 mL RhB (600 ppm) solution, 1 mL dosage of 30 wt% H_2O_2 at 313 K with initial pH = 2.5).

The isoelectric point of Fe_3O_4-Cu/CA catalyst lies at pH range between 2.5 and 3.0. At pH of 2.5, the catalyst shows positive Z potential of 7.46. Whereas, RhB has positive charge polarity within a pH value below 3.0 (it tends to be protonated at a pH below 3.0), which is attributed to its quaternary ammonium with cationic character . It indicates that coulombic force between the Fe_3O_4-Cu/CA catalyst and RhB substrate shows mutual exclusion, which does not favor the adsorption of RhB on the catalyst surface.

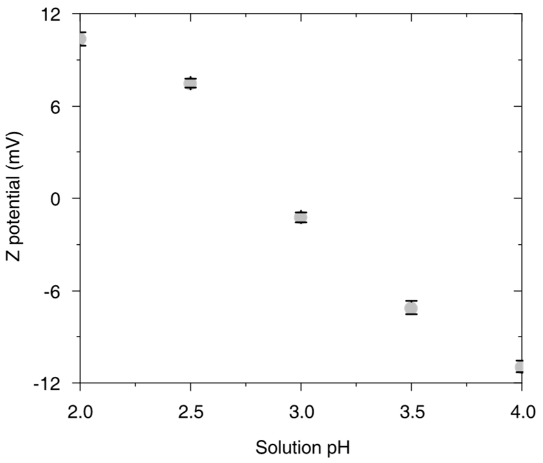

Figure S9 The zeta potential of Fe_3O_4-Cu/CA under different pH conditions.

The data on leached iron and copper at different pHs are also measured and listed in the revised supporting information. We designed a large-scale reaction under different pHs within 30 min for degrading 100 mL of 600 ppm RhB with a catalyst dosage of 10 mg and 9.8 mmol of H_2O_2, at 40 ºC. The total leached iron concentration is 3.18 mg/L at pH of 2.0, 6.19 mg/L at pH of 2.5, 0.58 mg/L at pH of 3.0, 0.75 mg/L at pH of 3.5, respectively. Meanwhile, the total leached copper concentration is 8.38 mg/L at pH of 2.0, 12.82 mg/L at pH of 2.5, 8.2 mg/L at pH of 3.0, 7.42 mg/L at pH of 3.5, respectively. The leached metal comes to the maximum at pH of 2.5, and the degradation of RhB reaches almost in our test.

To account for the possible synergistic effect of the presence of copper. Fenton-like reaction of degrading RhB should be performed by adding either catalyst or iron salts in the absence of copper in system. The amount of added iron is in accordance with the total leached iron concentration of 6.19 mg/L at a pH of 2.5. As a result, 1.0 mg $FeSO_4 \cdot 7H_2O$ and 3.0 mg $Fe(NO_3)_3 \cdot 9H_2O$ is added into the Fenton-like reaction of degradation 100 mL of 600 ppm RhB with adding 9.8 mmol of H_2O_2, at 40 ºC. The RhB degradation ratio has been listed in Table S1. The experimental results show that Fe_3O_4-Cu/CA shows more active catalytic performance than homogeneous Fenton reaction. This reveals the RhB degradation reaction is promoted by the possible synergistic effect of Fe-Cu bimetallic sites in Fe_3O_4-Cu/CA.

Table S1 The Fenton-like reaction of degrading 100 mL of 600 ppm RhB with adding 9.8 mmol of H_2O_2, at 40 ºC under different conditions.

**Catalyst**	**dosage**	**RhB degradation ratio within 10 min**	**RhB degradation ratio within 20 min**	**RhB degradation ratio within 30 min**
Fe_3O_4-Cu/CA	10 mg	93.4%	99.3%	100.0%
$FeSO_4$ • 7H2O/ $Fe(NO_3)_3$ • 9H2O	1 mg/ 3 mg	42.6%	63.2%	83.0%
